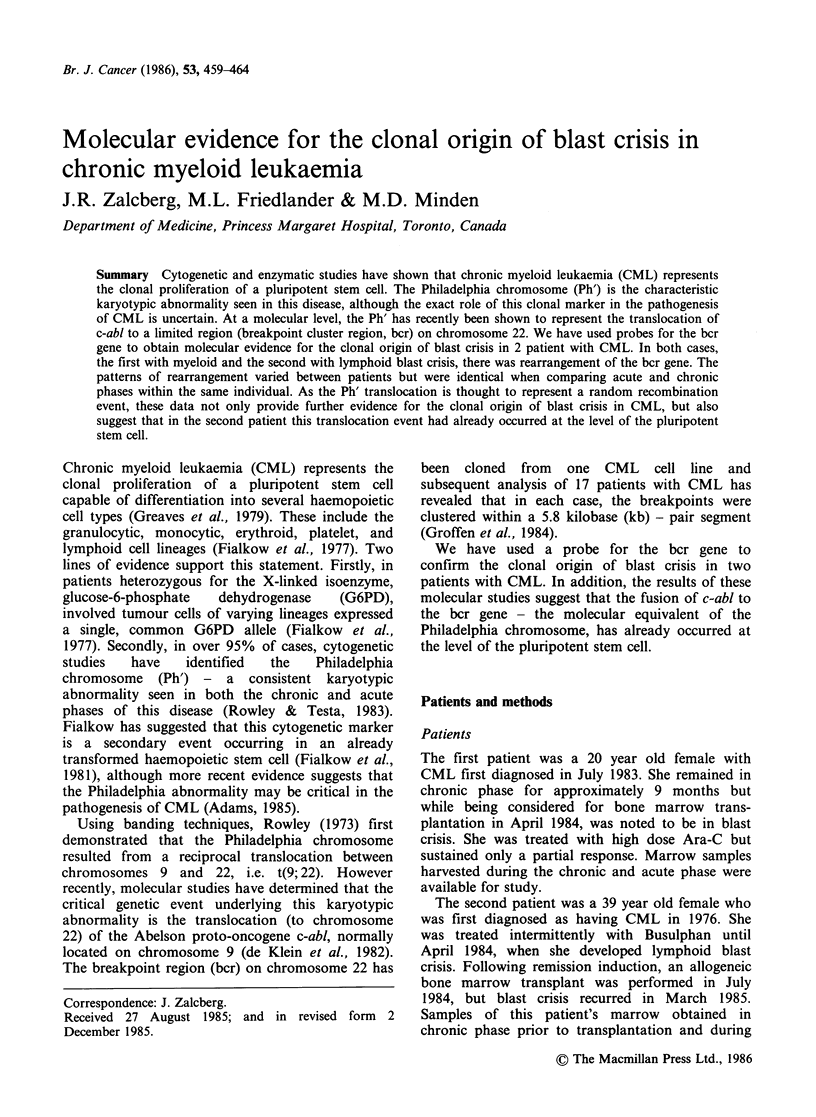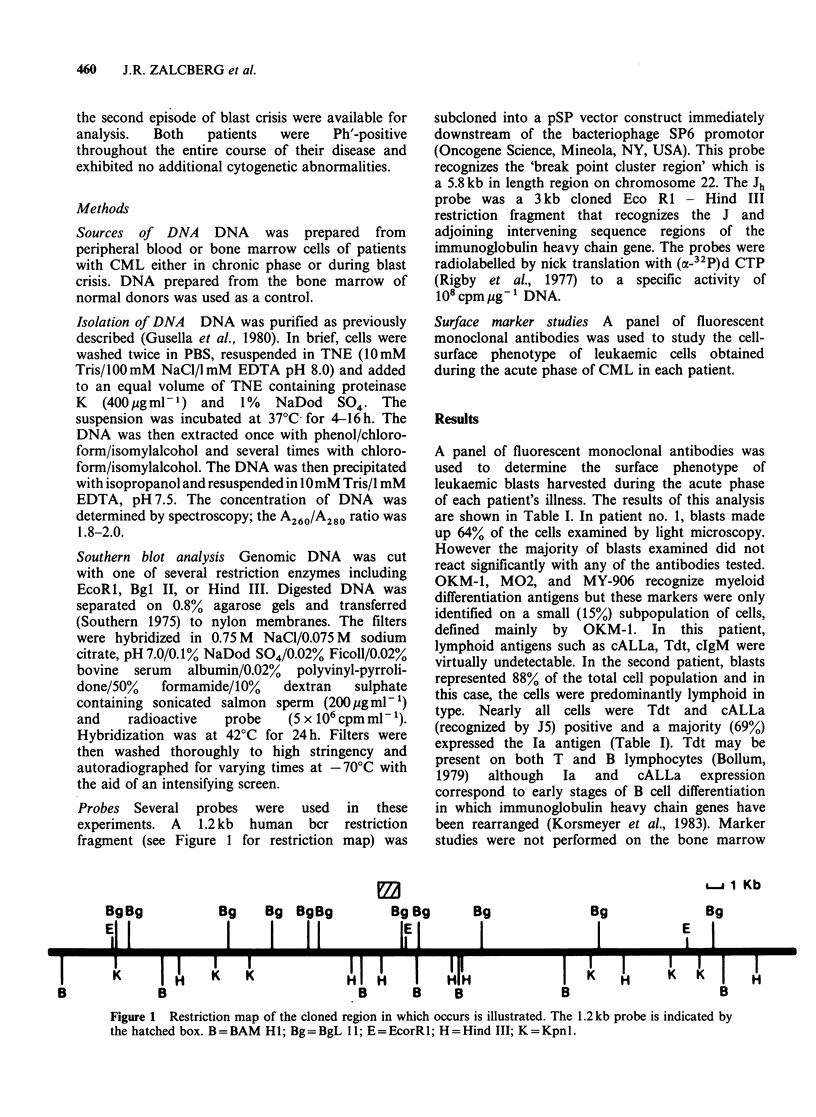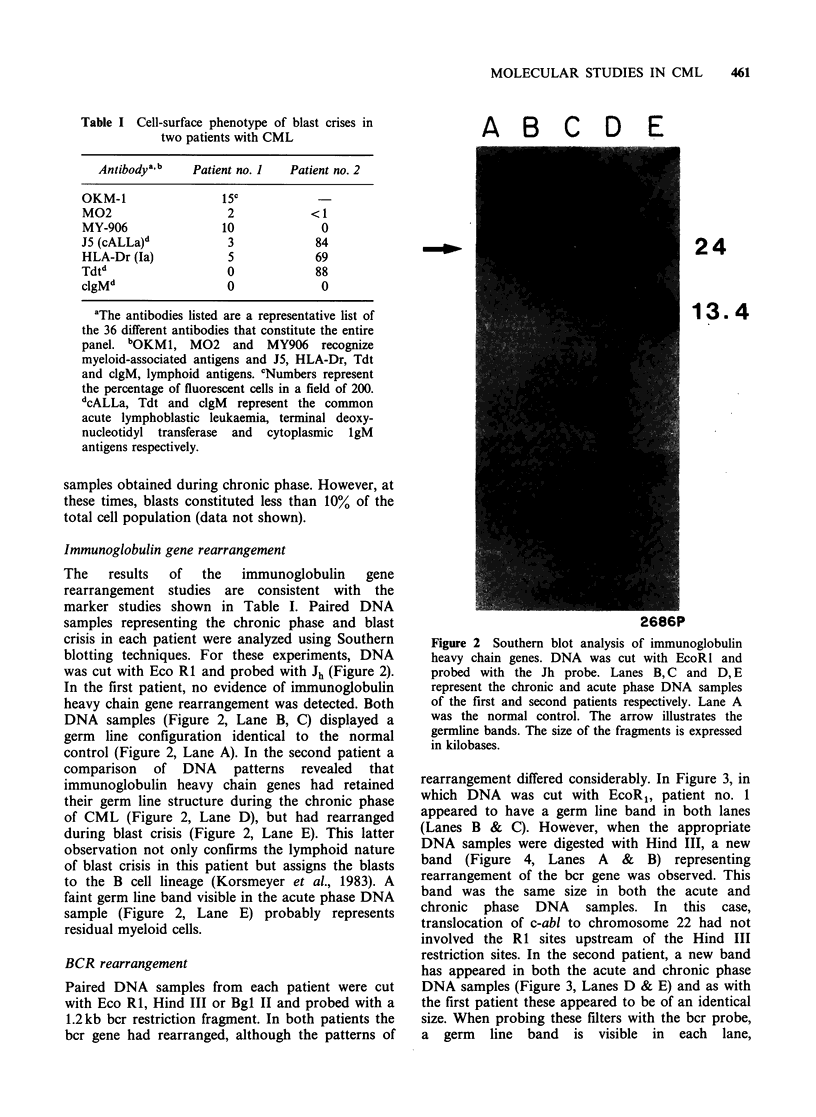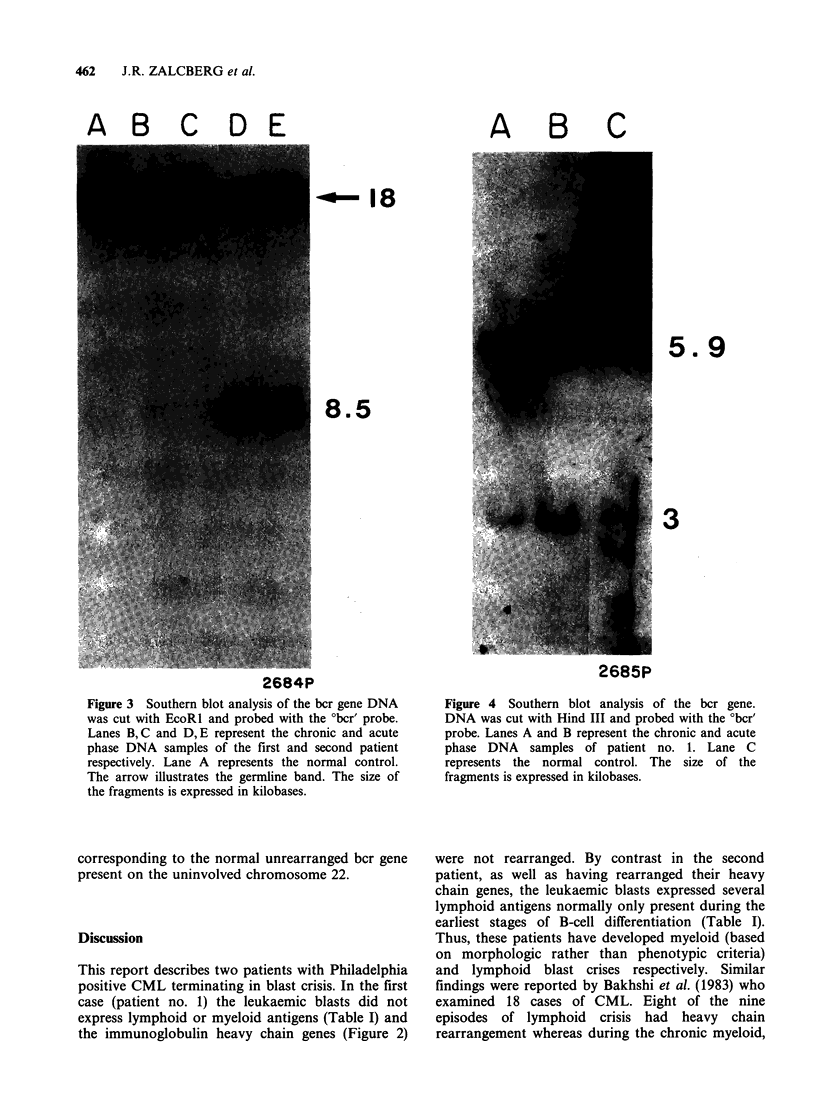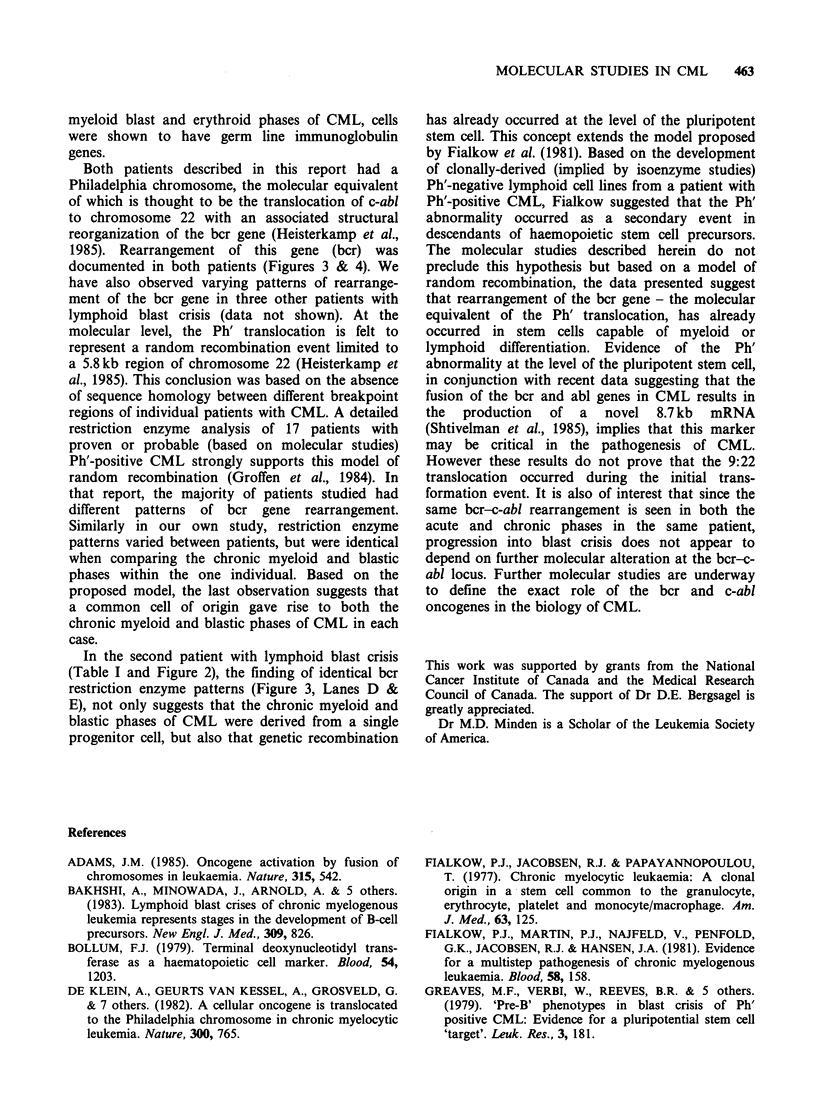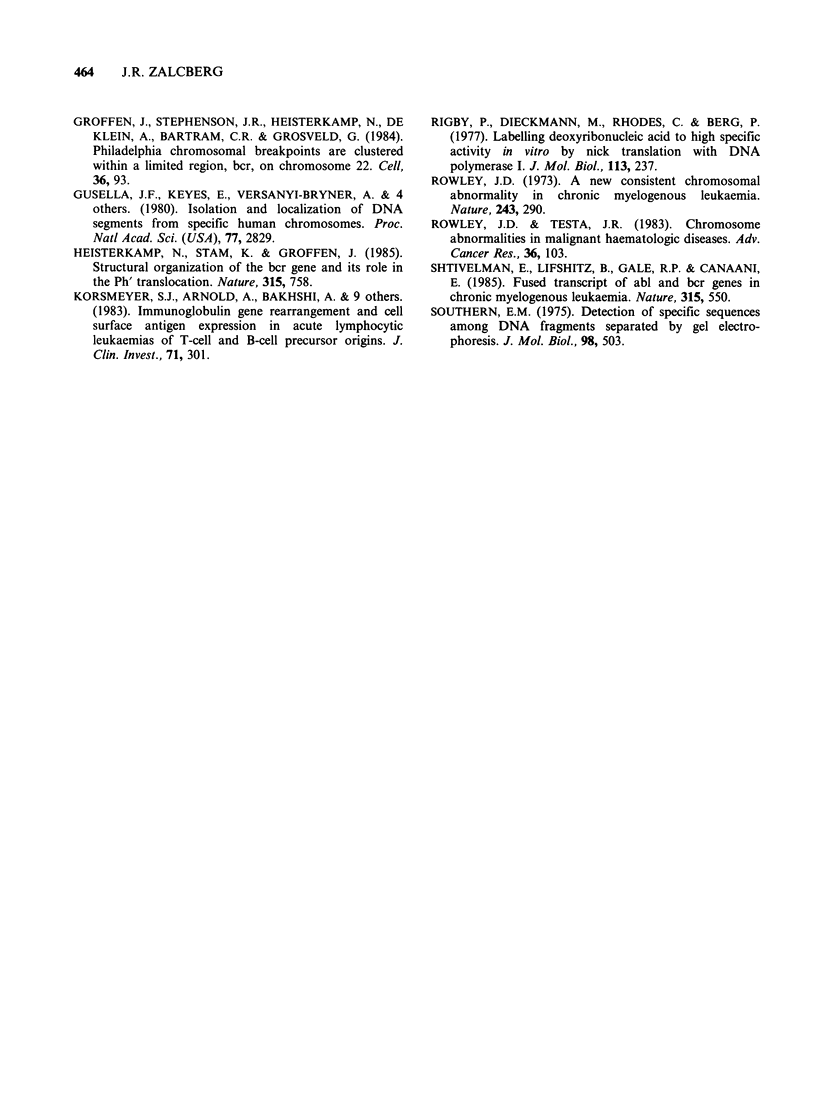# Molecular evidence for the clonal origin of blast crisis in chronic myeloid leukaemia.

**DOI:** 10.1038/bjc.1986.73

**Published:** 1986-04

**Authors:** J. R. Zalcberg, M. L. Friedlander, M. D. Minden

## Abstract

**Images:**


*Br. J. Cancer* (1986), **53**, 459–464

### Molecular evidence for the clonal origin of blast crisis in chronic myeloid leukaemia

J.R. Zalcberg, M.L. Friedlander & M.D. Minden

*Department of Medicine, Princess Margaret Hospital, Toronto, Canada*

**Summary** Cytogenetic and enzymatic studies have shown that chronic myeloid leukaemia (CML) represents the clonal proliferation of a pluripotent stem cell. The Philadelphia chromosome (Ph′) is the characteristic karyotypic abnormality seen in this disease, although the exact role of this clonal marker in the pathogenesis of CML is uncertain. At a molecular level, the Ph′ has recently been shown to represent the translocation of c-*abl* to a limited region (breakpoint cluster region, bcr) on chromosome 22. We have used probes for the bcr gene to obtain molecular evidence for the clonal origin of blast crisis in 2 patient with CML. In both cases, the first with myeloid and the second with lymphoid blast crisis, there was rearrangement of the bcr gene. The patterns of rearrangement varied between patients but were identical when comparing acute and chronic phases within the same individual. As the Ph′ translocation is thought to represent a random recombination event, these data not only provide further evidence for the clonal origin of blast crisis in CML, but also suggest that in the second patient this translocation event had already occurred at the level of the pluripotent stem cell.

Chronic myeloid leukaemia (CML) represents the clonal proliferation of a pluripotent stem cell capable of differentiation into several haemopoietic cell types (Greaves *et al.,* 1979). These include the granulocytic, monocytic, erythroid, platelet, and lymphoid cell lineages (Fialkow *et al.,* 1977). Two lines of evidence support this statement. Firstly, in patients heterozygous for the X-linked isoenzyme, glucose-6-phosphate dehydrogenase (G6PD), involved tumour cells of varying lineages expressed a single, common G6PD allele (Fialkow *et al.,* 1977). Secondly, in over 95% of cases, cytogenetic studies have identified the Philadelphia chromosome (Ph′) – a consistent karyotypic abnormality seen in both the chronic and acute phases of this disease (Rowley & Testa, 1983). Fialkow has suggested that this cytogenetic marker is a secondary event occurring in an already transformed haemopoietic stem cell (Fialkow *et al.,* 1981), although more recent evidence suggests that the Philadelphia abnormality may be critical in the pathogenesis of CML (Adams, 1985).

Using banding techniques, Rowley (1973) first demonstrated that the Philadelphia chromosome resulted from a reciprocal translocation between chromosomes 9 and 22, i.e. t(9;22). However recently, molecular studies have determined that the critical genetic event underlying this karyotypic abnormality is the translocation (to chromosome 22) of the Abelson proto-oncogene c-*abl*, normally located on chromosome 9 (de Klein *et al.,* 1982). The breakpoint region (bcr) on chromosome 22 has been cloned from one CML cell line and subsequent analysis of 17 patients with CML has revealed that in each case, the breakpoints were clustered within a 5.8 kilobase (kb) – pair segment (Groffen *et al.,* 1984).

We have used a probe for the bcr gene to confirm the clonal origin of blast crisis in two patients with CML. In addition, the results of these molecular studies suggest that the fusion of *c-abl* to the bcr gene – the molecular equivalent of the Philadelphia chromosome, has already occurred at the level of the pluripotent stem cell.

Correspondence: J. Zalcberg.
Received 27 August 1985; and in revised form 2 December 1985.

### Patients and methods

### *Patients*

The first patient was a 20 year old female with CML first diagnosed in July 1983. She remained in chronic phase for approximately 9 months but while being considered for bone marrow transplantation in April 1984, was noted to be in blast crisis. She was treated with high dose Ara-C but sustained only a partial response. Marrow samples harvested during the chronic and acute phase were available for study.

The second patient was a 39 year old female who was first diagnosed as having CML in 1976. She was treated intermittently with Busulphan until April 1984, when she developed lymphoid blast crisis. Following remission induction, an allogeneic bone marrow transplant was performed in July 1984, but blast crisis recurred in March 1985. Samples of this patient's marrow obtained in chronic phase prior to transplantation and during

© The Macmillan Press Ltd., 1986

the second episode of blast crisis were available for analysis. Both patients were Ph′-positive throughout the entire course of their disease and exhibited no additional cytogenetic abnormalities.

### *Methods*

*Sources of DNA* DNA was prepared from peripheral blood or bone marrow cells of patients with CML either in chronic phase or during blast crisis. DNA prepared from the bone marrow of normal donors was used as a control.

*Isolation of DNA* DNA was purified as previously described (Gusella *et al.*, 1980). In brief, cells were washed twice in PBS, resuspended in TNE (10 mM Tris/100 mM NaCl/1 mM EDTA pH 8.0) and added to an equal volume of TNE containing proteinase K (400 $\mu g ml^{-1}$) and 1% NaDod $SO_4$. The suspension was incubated at 37°C for 4–16 h. The DNA was then extracted once with phenol/chloroform/isomylalcohol and several times with chloroform/isomylalcohol. The DNA was then precipitated with isopropanol and resuspended in 10 mM Tris/1 mM EDTA, pH 7.5. The concentration of DNA was determined by spectroscopy; the $A_{260}/A_{280}$ ratio was 1.8–2.0.

*Southern blot analysis* Genomic DNA was cut with one of several restriction enzymes including EcoR1, Bgl II, or Hind III. Digested DNA was separated on 0.8% agarose gels and transferred (Southern 1975) to nylon membranes. The filters were hybridized in 0.75 M NaCl/0.075 M sodium citrate, pH 7.0/0.1% NaDod $SO_4$/0.02% Ficoll/0.02% bovine serum albumin/0.02% polyvinyl-pyrrolidone/50% formamide/10% dextran sulphate containing sonicated salmon sperm (200 $\mu g ml^{-1}$) and radioactive probe ($5 \times 10^6$ cpm $ml^{-1}$). Hybridization was at 42°C for 24 h. Filters were then washed thoroughly to high stringency and autoradiographed for varying times at −70°C with the aid of an intensifying screen.

*Probes* Several probes were used in these experiments. A 1.2 kb human bcr restriction fragment (see Figure 1 for restriction map) was subcloned into a pSP vector construct immediately downstream of the bacteriophage SP6 promotor (Oncogene Science, Mineola, NY, USA). This probe recognizes the 'break point cluster region' which is a 5.8 kb in length region on chromosome 22. The $J_h$ probe was a 3 kb cloned Eco R1 – Hind III restriction fragment that recognizes the J and adjoining intervening sequence regions of the immunoglobulin heavy chain gene. The probes were radiolabelled by nick translation with ($\alpha$-$^{32}P$)d CTP (Rigby *et al.*, 1977) to a specific activity of $10^8$ cpm $\mu g^{-1}$ DNA.

*Surface marker studies* A panel of fluorescent monoclonal antibodies was used to study the cell-surface phenotype of leukaemic cells obtained during the acute phase of CML in each patient.

### Results

A panel of fluorescent monoclonal antibodies was used to determine the surface phenotype of leukaemic blasts harvested during the acute phase of each patient's illness. The results of this analysis are shown in Table I. In patient no. 1, blasts made up 64% of the cells examined by light microscopy. However the majority of blasts examined did not react significantly with any of the antibodies tested. OKM-1, MO2, and MY-906 recognize myeloid differentiation antigens but these markers were only identified on a small (15%) subpopulation of cells, defined mainly by OKM-1. In this patient, lymphoid antigens such as cALLa, Tdt, cIgM were virtually undetectable. In the second patient, blasts represented 88% of the total cell population and in this case, the cells were predominantly lymphoid in type. Nearly all cells were Tdt and cALLa (recognized by J5) positive and a majority (69%) expressed the Ia antigen (Table I). Tdt may be present on both T and B lymphocytes (Bollum, 1979) although Ia and cALLa expression correspond to early stages of B cell differentiation in which immunoglobulin heavy chain genes have been rearranged (Korsmeyer *et al.*, 1983). Marker studies were not performed on the bone marrow

**Figure 1** Restriction map of the cloned region in which occurs is illustrated. The 1.2 kb probe is indicated by the hatched box. B = BAM H1; Bg = BgL 11; E = EcorR1; H = Hind III; K = Kpn1.

**Table I** Cell-surface phenotype of blast crises in two patients with CML

| *Antibody*[a,b] | *Patient no. 1* | *Patient no. 2* |
|---|---|---|
| OKM-1 | 15[c] | — |
| MO2 | 2 | <1 |
| MY-906 | 10 | 0 |
| J5 (cALLa)[d] | 3 | 84 |
| HLA-Dr (Ia) | 5 | 69 |
| Tdt[d] | 0 | 88 |
| clgM[d] | 0 | 0 |

[a]The antibodies listed are a representative list of the 36 different antibodies that constitute the entire panel. [b]OKM1, MO2 and MY906 recognize myeloid-associated antigens and J5, HLA-Dr, Tdt and clgM, lymphoid antigens. [c]Numbers represent the percentage of fluorescent cells in a field of 200. [d]cALLa, Tdt and clgM represent the common acute lymphoblastic leukaemia, terminal deoxynucleotidyl transferase and cytoplasmic 1gM antigens respectively.

samples obtained during chronic phase. However, at these times, blasts constituted less than 10% of the total cell population (data not shown).

### *Immunoglobulin gene rearrangement*

The results of the immunoglobulin gene rearrangement studies are consistent with the marker studies shown in Table I. Paired DNA samples representing the chronic phase and blast crisis in each patient were analyzed using Southern blotting techniques. For these experiments, DNA was cut with Eco R1 and probed with $J_h$ (Figure 2). In the first patient, no evidence of immunoglobulin heavy chain gene rearrangement was detected. Both DNA samples (Figure 2, Lane B, C) displayed a germ line configuration identical to the normal control (Figure 2, Lane A). In the second patient a comparison of DNA patterns revealed that immunoglobulin heavy chain genes had retained their germ line structure during the chronic phase of CML (Figure 2, Lane D), but had rearranged during blast crisis (Figure 2, Lane E). This latter observation not only confirms the lymphoid nature of blast crisis in this patient but assigns the blasts to the B cell lineage (Korsmeyer *et al.*, 1983). A faint germ line band visible in the acute phase DNA sample (Figure 2, Lane E) probably represents residual myeloid cells.

### *BCR rearrangement*

Paired DNA samples from each patient were cut with Eco R1, Hind III or Bgl II and probed with a 1.2 kb bcr restriction fragment. In both patients the bcr gene had rearranged, although the patterns of

**Figure 2** Southern blot analysis of immunoglobulin heavy chain genes. DNA was cut with EcoR1 and probed with the Jh probe. Lanes B, C and D, E represent the chronic and acute phase DNA samples of the first and second patients respectively. Lane A was the normal control. The arrow illustrates the germline bands. The size of the fragments is expressed in kilobases.

rearrangement differed considerably. In Figure 3, in which DNA was cut with $EcoR_1$, patient no. 1 appeared to have a germ line band in both lanes (Lanes B & C). However, when the appropriate DNA samples were digested with Hind III, a new band (Figure 4, Lanes A & B) representing rearrangement of the bcr gene was observed. This band was the same size in both the acute and chronic phase DNA samples. In this case, translocation of c-*abl* to chromosome 22 had not involved the R1 sites upstream of the Hind III restriction sites. In the second patient, a new band has appeared in both the acute and chronic phase DNA samples (Figure 3, Lanes D & E) and as with the first patient these appeared to be of an identical size. When probing these filters with the bcr probe, a germ line band is visible in each lane,

**Figure 3** Southern blot analysis of the bcr gene DNA was cut with EcoR1 and probed with the °bcr′ probe. Lanes B, C and D, E represent the chronic and acute phase DNA samples of the first and second patient respectively. Lane A represents the normal control. The arrow illustrates the germline band. The size of the fragments is expressed in kilobases.

**Figure 4** Southern blot analysis of the bcr gene. DNA was cut with Hind III and probed with the °bcr′ probe. Lanes A and B represent the chronic and acute phase DNA samples of patient no. 1. Lane C represents the normal control. The size of the fragments is expressed in kilobases.

corresponding to the normal unrearranged bcr gene present on the uninvolved chromosome 22.

### Discussion

This report describes two patients with Philadelphia positive CML terminating in blast crisis. In the first case (patient no. 1) the leukaemic blasts did not express lymphoid or myeloid antigens (Table I) and the immunoglobulin heavy chain genes (Figure 2) were not rearranged. By contrast in the second patient, as well as having rearranged their heavy chain genes, the leukaemic blasts expressed several lymphoid antigens normally only present during the earliest stages of B-cell differentiation (Table I). Thus, these patients have developed myeloid (based on morphologic rather than phenotypic criteria) and lymphoid blast crises respectively. Similar findings were reported by Bakhshi *et al.* (1983) who examined 18 cases of CML. Eight of the nine episodes of lymphoid crisis had heavy chain rearrangement whereas during the chronic myeloid, myeloid blast and erythroid phases of CML, cells were shown to have germ line immunoglobulin genes.

Both patients described in this report had a Philadelphia chromosome, the molecular equivalent of which is thought to be the translocation of c-*abl* to chromosome 22 with an associated structural reorganization of the bcr gene (Heisterkamp *et al.*, 1985). Rearrangement of this gene (bcr) was documented in both patients (Figures 3 & 4). We have also observed varying patterns of rearrangement of the bcr gene in three other patients with lymphoid blast crisis (data not shown). At the molecular level, the Ph′ translocation is felt to represent a random recombination event limited to a 5.8 kb region of chromosome 22 (Heisterkamp *et al.*, 1985). This conclusion was based on the absence of sequence homology between different breakpoint regions of individual patients with CML. A detailed restriction enzyme analysis of 17 patients with proven or probable (based on molecular studies) Ph′-positive CML strongly supports this model of random recombination (Groffen *et al.*, 1984). In that report, the majority of patients studied had different patterns of bcr gene rearrangement. Similarly in our own study, restriction enzyme patterns varied between patients, but were identical when comparing the chronic myeloid and blastic phases within the one individual. Based on the proposed model, the last observation suggests that a common cell of origin gave rise to both the chronic myeloid and blastic phases of CML in each case.

In the second patient with lymphoid blast crisis (Table I and Figure 2), the finding of identical bcr restriction enzyme patterns (Figure 3, Lanes D & E), not only suggests that the chronic myeloid and blastic phases of CML were derived from a single progenitor cell, but also that genetic recombination has already occurred at the level of the pluripotent stem cell. This concept extends the model proposed by Fialkow *et al.* (1981). Based on the development of clonally-derived (implied by isoenzyme studies) Ph′-negative lymphoid cell lines from a patient with Ph′-positive CML, Fialkow suggested that the Ph′ abnormality occurred as a secondary event in descendants of haemopoietic stem cell precursors. The molecular studies described herein do not preclude this hypothesis but based on a model of random recombination, the data presented suggest that rearrangement of the bcr gene – the molecular equivalent of the Ph′ translocation, has already occurred in stem cells capable of myeloid or lymphoid differentiation. Evidence of the Ph′ abnormality at the level of the pluripotent stem cell, in conjunction with recent data suggesting that the fusion of the bcr and abl genes in CML results in the production of a novel 8.7 kb mRNA (Shtivelman *et al.*, 1985), implies that this marker may be critical in the pathogenesis of CML. However these results do not prove that the 9:22 translocation occurred during the initial transformation event. It is also of interest that since the same bcr–c-*abl* rearrangement is seen in both the acute and chronic phases in the same patient, progression into blast crisis does not appear to depend on further molecular alteration at the bcr–c-*abl* locus. Further molecular studies are underway to define the exact role of the bcr and c-*abl* oncogenes in the biology of CML.

This work was supported by grants from the National Cancer Institute of Canada and the Medical Research Council of Canada. The support of Dr D.E. Bergsagel is greatly appreciated.

Dr M.D. Minden is a Scholar of the Leukemia Society of America.

**References**

ADAMS, J.M. (1985). Oncogene activation by fusion of chromosomes in leukaemia. *Nature,* **315,** 542.

BAKHSHI, A., MINOWADA, J., ARNOLD, A. & 5 others. (1983). Lymphoid blast crises of chronic myelogenous leukemia represents stages in the development of B-cell precursors. *New Engl. J. Med.,* **309,** 826.

BOLLUM, F.J. (1979). Terminal deoxynucleotidyl transferase as a haematopoietic cell marker. *Blood,* **54,** 1203.

DE KLEIN, A., GEURTS VAN KESSEL, A., GROSVELD, G. & 7 others. (1982). A cellular oncogene is translocated to the Philadelphia chromosome in chronic myelocytic leukemia. *Nature,* **300,** 765.

FIALKOW, P.J., JACOBSEN, R.J. & PAPAYANNOPOULOU, T. (1977). Chronic myelocytic leukaemia: A clonal origin in a stem cell common to the granulocyte, erythrocyte, platelet and monocyte/macrophage. *Am. J. Med.,* **63,** 125.

FIALKOW, P.J., MARTIN, P.J., NAJFELD, V., PENFOLD, G.K., JACOBSEN, R.J. & HANSEN, J.A. (1981). Evidence for a multistep pathogenesis of chronic myelogenous leukaemia. *Blood,* **58,** 158.

GREAVES, M.F., VERBI, W., REEVES, B.R. & 5 others. (1979). 'Pre-B' phenotypes in blast crisis of Ph′ positive CML: Evidence for a pluripotential stem cell 'target'. *Leuk. Res.,* **3,** 181.

GROFFEN, J., STEPHENSON, J.R., HEISTERKAMP, N., DE KLEIN, A., BARTRAM, C.R. & GROSVELD, G. (1984). Philadelphia chromosomal breakpoints are clustered within a limited region, bcr, on chromosome 22. *Cell*, **36**, 93.

GUSELLA, J.F., KEYES, E., VERSANYI-BRYNER, A. & 4 others. (1980). Isolation and localization of DNA segments from specific human chromosomes. *Proc. Natl Acad. Sci.* (*USA*), **77**, 2829.

HEISTERKAMP, N., STAM, K. & GROFFEN, J. (1985). Structural organization of the bcr gene and its role in the Ph′ translocation. *Nature*, **315**, 758.

KORSMEYER, S.J., ARNOLD, A., BAKHSHI, A. & 9 others. (1983). Immunoglobulin gene rearrangement and cell surface antigen expression in acute lymphocytic leukaemias of T-cell and B-cell precursor origins. *J. Clin. Invest.*, **71**, 301.

RIGBY, P., DIECKMANN, M., RHODES, C. & BERG, P. (1977). Labelling deoxyribonucleic acid to high specific activity *in vitro* by nick translation with DNA polymerase I. *J. Mol. Biol.*, **113**, 237.

ROWLEY, J.D. (1973). A new consistent chromosomal abnormality in chronic myelogenous leukaemia. *Nature*, **243**, 290.

ROWLEY, J.D. & TESTA, J.R. (1983). Chromosome abnormalities in malignant haematologic diseases. *Adv. Cancer Res.*, **36**, 103.

SHTIVELMAN, E., LIFSHITZ, B., GALE, R.P. & CANAANI, E. (1985). Fused transcript of abl and bcr genes in chronic myelogenous leukaemia. *Nature*, **315**, 550.

SOUTHERN, E.M. (1975). Detection of specific sequences among DNA fragments separated by gel electrophoresis. *J. Mol. Biol.*, **98**, 503.